# Rapamycin combined with osimertinib alleviated non-small cell lung cancer by regulating the PARP, Akt/mTOR, and MAPK/ERK signaling pathways

**DOI:** 10.3389/fmolb.2025.1548810

**Published:** 2025-03-07

**Authors:** Qingrong Ma, Kai Chen, Haiping Xiao

**Affiliations:** Thoracic and Cardiac Surgery, The First Affiliated Hospital of Guangdong Pharmaceutical University, Guangzhou, Guangdong, China

**Keywords:** rapamycin, osimertinib, non-small cell lung cancer, PARP signaling pathway, Akt/mTOR signaling pathway, MAPK/ERK signaling pathway

## Abstract

**Backgrounds:**

Non-small cell lung cancer (NSCLC), one kind of common malignant tumor, is accompanied by high morbidity and mortality. The effects and related mechanisms of rapamycin (Rapa) combined with osimertinib (Osi) in treating NSCLC are still unclear. Therefore, this study aims to investigate the effects and related mechanisms of Rapa combined with Osi on NSCLC.

**Methods:**

In A549 and PC-9 cells, the Cell Counting Kit-8 (CCK-8) assay was used to select the optimal administrative concentrations of Rapa and Osi and evaluate the cell viability. The Transwell assay and flow cytometry were used to determine the migration, cell cycle, apoptosis, and the level of Reactive Oxygen Species (ROS), respectively. The protein and mRNA expression level of Matrix Metalloproteinase-9 (MMP9), Caspase-3, Microtubule-Associated Protein 1 Light Chain 3 II/I (LC3 II/I), beclin1, Sequestosome 1 (p62), Poly (ADP-ribose) Polymerase (PARP), Mitogen-Activated Protein Kinase (MAPK), Extracellular Signal-Regulated Kinase (ERK), Protein Kinase B (Akt), and Mammalian Target of Rapamycin (mTOR) was determined by Western blot and Quantitative Reverse Transcription Polymerase Chain Reaction (qRT-PCR).

**Results:**

The optimal administrative concentrations of Rapa and Osi were 0.5 μM and 1 μM, respectively. Rapamycin combined with Osimertinib significantly decreased the viability of cells, the quantity of migrated cells, the levels of ROS, as well as the mRNA and protein expression levels of MMP9 and p62, Caspase-3, LC3 II/I, beclin1. The combination of the two drugs is markedly more effective than the use of drugs alone.

**Conclusion:**

In conclusion, the study demonstrated that Rapamycin combined with Osimertinib can inhibit the cell migration, regulate the cell cycle, promote the autophagy and apoptosis, increase the ROS level and regulate the PARP, MAPK/EKR, and Akt/mTOR pathways in A549 and PC-9 cells, providing a novel theoretical basis for their clinical treatment of NSCLC.

## 1 Introduction

Lung cancer is the most prevalent malignancy and a leading cause of cancer-related mortality worldwide. According to a study on global cancer epidemiology, lung cancer is the primary cause of cancer deaths, with non-small cell lung cancer (NSCLC) accounting for approximately 80% of all lung cancer cases (Bray et al., 2024). NSCLC, unlike small cell carcinomas, is characterized by slower growth, early stealth, and a tendency to be diagnosed at an advanced stage. Consequently, the treatment is more challenging, and the 5-year survival rate for most patients after diagnosis is only 19% (Wang et al., 2021). However, drug resistance is inevitable due to tumor heterogeneity, which drives the development and exploration of more efficient and less toxic drugs. The primary oncogenic driver in NSCLC is Epidermal Growth Factor Receptor (EGFR) mutation, whose classic mutations, such as exon 19 deletion and exon 21 mutation, are key targets for NSCLC therapy. First- and second-gen EGFR-TKIs show over 60% response in classic EGFR mutations, improving survival, but resistance emerges after 9–14 months, with 50% showing T790M mutation in exon 20 (Harrison et al., 2020). Osimertinib, as the first third-generation EGFR-TKI for treating T790M resistance mutations, was recommended as the standard first-line treatment for advanced or metastatic NSCLC patients with EGFR mutations based on its remarkable efficacy and manageable safety (Fu et al., 2022). However, the resistance of Osi treatment is endlessly developing, which limits the long-term treatment of NSCLC. Therefore, developing effective treatments to overcome the Osi resistance of NSCLC become an urgent need and get more concentrate. Rapamycin’s mammalian target (mTOR) is a serine/threonine protein kinase, and the signaling pathways associated with mTOR are complex and widely involved in the development of tumors (Marafie et al., 2024). The biological functions of tumor cells are affected when mTOR signaling is activated, such as apoptosis and autophagy (Tian et al., 2019). Rapamycin (Rapa), an mTOR inhibitor (mTORi), is commonly used to treat various types of cancer (Chen et al., 2023). Experimental results demonstrate that Rapa can induce apoptosis in lung cancer NCI-H446 cells and inhibit cell migration and invasion capabilities (Cao and Ma, 2020). Additionally, the combination of Rapa with other drugs can regulate the invasive and migratory abilities of cancer cells (Shen et al., 2018). Preclinical studies have confirmed the efficacy of Rapa in inhibiting the growth and proliferation of cancer cells (Magaway et al., 2019). Following the development and application of mTOR inhibitors, the first generation of mTOR inhibitors such as temsirolimus and everolimus emerged as Rapa derivatives. Everolimus combined Pan-EGRF Inhibitors also was studied in NSCLC. The result showed that the migration, invasion, adhesion, tumor perimeter, and mesenchymal phenotype were increased in the H292 KRAS mutated cells, and everolimus restored sensibility and improved cytotoxicity of EGFR inhibitors in the KRAS mutant NSCLC cell lines (da Silva-Oliveira et al., 2022). The Food and Drug Administration approved them for the clinical treatment of advanced renal cell carcinoma in 2007 and 2009, respectively, but their clinical outcomes have been less than satisfactory, with acquired resistance occurring in patients (Anandappa et al., 2010; Hamieh et al., 2018). Therefore, understanding the possible mechanisms of mTORi resistance and devising new strategies to enhance the clinical benefits of mTORi is of great importance.

The ERK is a serine/threonine protein kinase, and the MAPK/ERK signaling cascade is a phylogenetically conserved signal transduction pathway (Guo et al., 2020). The MAPK/ERK pathway is abnormally activated in over 30% of human cancers playing a significant role in malignant tumorigenesis (Bica et al., 2023). Recent studies have confirmed the significance of ERK in the biology of cancer cells. Specifically, the suppression of ERK and p38 phosphorylation canregulate the proliferation and apoptosis of breast cancer cells. Conversely, the activation of ERK phosphorylation modulates metastasis-associated genes and contributes to the invasion, migration, and proliferation of hepatocellular carcinoma cells (Zhang et al., 2016; Xie et al., 2021). Autophagy is an important lysosome-dependent degradation pathway within cells for the removal of damaged or senescent organelles, and abnormal autophagy is closely associated with cellular damage and the development of cancer (Choi et al., 2020). The activation of MAPK/ERK and Akt/mTOR promotes the proliferative activity of cancer cells by regulating autophagy (Qian et al., 2023). In addition, the PARP pathway causes drug resistance in tumor cells by mainly participating in DNA damage repair, which affects the effectiveness of anti-tumor agents (Cai et al., 2023). Therefore, the MAPK/ERK, Akt/mTOR, and PARP signaling pathways are promising therapeutic strategies for human cancers.

In this study, we aimed to seek evidence for the rational combination therapy of Rapa and Osi in NSCLC cells based on the MAPK/ERK, Akt/mTOR, and PARP signaling pathways, providing a reference for further research on the combination therapy of Rapa and Osi in NSCLC.

## 2 Materials and methods

### 2.1 Cell culture and group

A549 and PC-9 cells purchased from Wuhan Pricella Biotechnology Co., Ltd. were cultured in RPMI 1640 medium (Gibco, Waltham, MA) supplemented with 10% fetal bovine serum (FBS) (Gibco, Waltham, MA) and 1% penicillin/streptomycin (Gibco, Waltham, MA) at 37°C in a humidified atmosphere containing 5% CO_2_. The cell experiments were conducted with A549 and PC-9 cell lines, divided into four groups: the control group, Rapa, Osi, and Rapa + Osi groups. The A549 and PC-9 cells in the control group were normally cultured. The A549 and PC-9 cells in the Rapa or Osi groups were treated with Rapa (0.5 μM, MedChemExpress, Princeton, NJ) or Osi (1 μM, MedChemExpress, Princeton, NJ) for 48 h, respectively. The A549 and PC-9 cells in the Rapa + Osi groups were treated with Rapa and Osi for 48 h. Each group was cultured under standardized conditions to ensure experimental uniformity.

### 2.2 Cell viability assay

The A549 and PC-9 cells were cultured to a confluent state and respectively subjected to treatment with Rapa (0, 0.05, 0.1, 0.5, 1, 5, 10 μM) and Osi (0, 0.1, 0.5, 1, 2, 5, 10 μM) for 24 or 48 h in 96-well plates. Following the treatment, a fresh medium was replaced, and 10 μL of CCK-8 reagent (Sigma-Aldrich, St. Louis, MO) was added to each well. The plates were then incubated for 2 h in a cell culture incubator, and absorbance was measured at 450 nm using a microplate reader (BioTek, Winooski, VT). Based on the results, appropriate concentrations of Rapa and Osi for subsequent experiments were selected. To further assess the impact of Rapa combined with Osi on the cell viability of A549 and PC-9 cells, they were treated with Rapa and Osi at the optimal concentration, either alone or in combination, for 48 h. The cell viability was determined using the CCK-8 assay following the manufacturer’s instructions. The absorbance was measured at 450 nm using a microplate reader.

### 2.3 Colony formation assay

The A549 and PC-9 cells were seeded in round cell culture plates at a density of 800 cells plates and treated with Rapa (0.5 μM) or/and Osi (1 μM), incubated at 37°C with 5% CO₂ for 14 days to allow colony formation. After incubation, the colonies were washed twice with phosphate-buffered saline (PBS), fixed with 4% paraformaldehyde for 15 min, and stained with 0.5% crystal violet (Sigma-Aldrich, St. Louis, MO) for 15 min at room temperature. The stained plates were washed with water and air-dried. The images were taken through an optical camera.

### 2.4 Cell migration assay

The migration of A549 and PC-9 cells was assessed using Transwell chambers (Corning, Corning, NY). Approximately 2 × 10^5^ cells in 100 μL of serum-free RPMI-1640 were seeded onto filter inserts in a 24-well plate, which was placed in RPMI-1640 with 10% FBS on the bottom of the membrane. Cells were incubated at 37 °C for 24 h. The membrane was then fixed with ethanol and treated with 3% crystal violet (Sigma-Aldrich, St. Louis, MO). The upper side of the membrane was removed and the cells remaining on the lower side were observed under a microscope. The cells were counted at four different locations in one field and viewed at ×400 magnification, and the mean value was calculated. The membranes were photographed under a microscope at ×100 magnification.

### 2.5 AO/EB staining

Cells were seeded in a six-well plate at a density of 2 × 10^5^ cells per well and incubated with Rapa (0.5 μM) or/and Osi (1 μM) for 48 h. After the indicated treatments, cells were washed twice with PBS and stained with a 1:1 mixture of acridine orange (AO) and ethidium bromide (EB) (maokangbio, China) at a final concentration of 5 μg/mL for 5 min in the dark. The stained cells were immediately observed under a fluorescence microscope (Olympus IX73, Japan) with an excitation wavelength of 488 nm and an emission wavelength of 515 nm. Viable cells appeared green, early apoptotic cells showed green fluorescence with chromatin condensation, late apoptotic cells exhibited orange-red fluorescence with fragmented nucleus, and necrotic cells appeared red with intact nucleus.

### 2.6 Cell cycle and apoptosis analysis

For cell cycle analysis, A549 and PC-9 cells were treated with the indicated drugs for 48 h, then fixed in 70% ethanol and stained with propidium iodide (PI) (Sigma-Aldrich, St. Louis, MO). For apoptosis analysis, A549 and PC-9 cells were treated with the indicated drugs for 48 h, then incubated with Annexin V-FITC and PI (BD Biosciences, San Jose, CA) for 15 min. The A549 and PC-9 cells were analyzed using a BD Accuri™ C6 Plus Flow Cytometer (BD Biosciences) to determine the cell cycle and apoptosis, and data were analyzed using FlowJo software (FlowJo LLC, Ashland, OR).

### 2.7 Reactive oxygen species (ROS) assay

Logarithmic phase A549 and PC-9 cells were seeded into a six-well plate at a density of 1 × 10^6^ cells/mL. After the cells adhered to the plate, the supernatant was discarded, and the cells were treated with the indicated drugs for 48 h. Then, DCFH-DA (Sigma-Aldrich, St. Louis, MO) was diluted in a serum-free medium at a ratio of 1:1,000 to a final concentration of 10 µM. The cell culture medium was removed, and the diluted DCFH-DA was added to the cells, which were then incubated in a 37°C incubator for 20 min. The cells were washed three times with serum-free medium to remove excessively uninternalized DCFH-DA. After washing with 1 mL PBS, the cells were centrifuged at 1,500 rpm for 5 min, and the supernatant was discarded. The cells were resuspended in 300 µL of PBS and then analyzed by a BD Accuri^™^ C6 Plus Flow Cytometer (BD Biosciences).

### 2.8 Quantitative real-time PCR (qRT-PCR)

The total RNAs in A549 and PC-9 cells were extracted and purified using the RNeasy Mini Kit (QIAGEN, Hilden, Germany). cDNA was synthesized using the QuantiTect Reverse Transcription Kit (QIAGEN, Hilden, Germany). qRT-PCR was performed using a QuantStudio® 3 Real-Time PCR system (Thermo Fisher Scientific, Waltham, MA) and SYBR Green Master Mix (Thermo Fisher Scientific, Waltham, MA). Gene expression levels were calculated using the 2^−ΔΔCT^ method, and GAPDH was used as the internal control.

### 2.9 Western blot analysis

The A549 and PC-9 cells were lysed in RIPA buffer with PMSF (Beyotime, China), and protein concentrations were measured using the BCA assay. Proteins were denatured at 1 μg/μL, boiled for 5 min, and stored at −80°C. Proteins were separated on 8%–12% Sodium Dodecyl Sulfate-Polyacrylamide Gel Electrophoresis (SDS-PAGE) gels and transferred to Polyvinylidene fluorid (PVDF, Millipore, Germany, XR732) membranes. Membranes were blocked in 5% milk, incubated with primary antibodies (including MAPK (1: 1,000, Abcam, Cambridge, United Kingdom), p-MAPK (1: 2000, Abcam, Cambridge, United Kingdom), ERK (1: 1,000, Abcam, Cambridge, United Kingdom), p-ERK (1: 2000, Abcam, Cambridge, United Kingdom), Akt (1: 1,000, Abcam, Cambridge, United Kingdom), p-Akt (1: 2000, Abcam, Cambridge, United Kingdom), mTOR (1: 1,000, Abcam, Cambridge, United Kingdom), p-mTOR (1: 2000, Abcam, Cambridge, United Kingdom), PARP (1: 1,000, Abcam, Cambridge, United Kingdom), cleaved PARP (1: 2000, Abcam, Cambridge, United Kingdom), caspase-3 (1: 1,000, Affinity, Nanjing, China), cleaved caspase-3 (1: 2000, Affinity, Nanjing, China), MMP-9 (1: 1,000, Affinity, Nanjing, China), p62 (1: 1,000, Affinity, Nanjing, China), LC3 I (1: 1,000, Affinity, Nanjing, China), LC3 II (1: 1,000, Affinity, Nanjing, China), beclin1 (1: 1,000, Affinity, Nanjing, China), and GAPDH (1: 5,000, Proteintech, Wuhan, China)) overnight at 4°C, washed, and incubated with HRP-conjugated goat anti-rabbit secondary antibodies (1: 2000, Proteintech, Wuhan, China) for 1 h at room temperature. After washing, ECL reagent (Beyotime, China) was applied, and membranes were imaged using the Image Quant LAS 4000C system. Band intensities were quantified using ImageJ, and experiments were repeated ≥3 times.

### 2.10 Statistical analysis

Data are presented as mean ± standard deviation of at least three independent experiments. Statistical significance was determined using Student’s t-test or one-way ANOVA followed by Tukey’s post-hoc test. A *p*-value of less than 0.05 was considered statistically significant.

## 3 Results

### 3.1 Rapamycin combined with osimertinib reduced the cell viability of A549 and PC-9 cells

**FIGURE 1:**
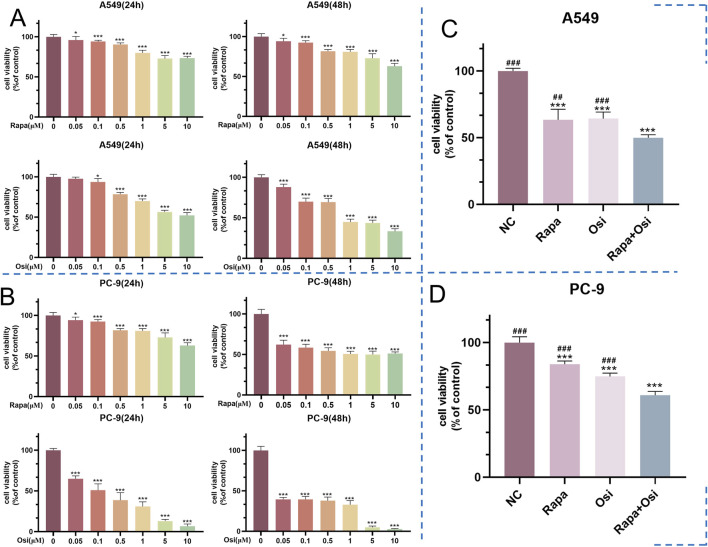
Effects of Rapa and Osi on the viability of A549 and PC-9 tumor cells at different concentrations and time points. The A549 and PC-9 cells were respectively subjected to treatment with Rapa (0, 0.05, 0.1, 0.5, 1, 5, 10 μM) and Osi (0, 0.1, 0.5, 1, 2, 5, 10 μM) for 24 or 48 h. The Rapa (0.5 μM) or/and Osi (1 μM) were selected to treat A549 and PC-9 cells for 48 h. **(A)** Cell viability of A549 cells treated with different concentrations of Rapa or/and Osi for 24 h and 48 h, respectively. **(B)** Cell viability of PC-9 cells treated with different concentrations of Rapa or/and Osi for 24 h and 48 h, respectively. **(C)** Combined effects of Rapa (0.5 μM) or/and Osi (1 μM) on the viability of A549 cells for 48 h. **(D)** Combined effects of Rapa (0.5 μM) or/and Osi (1 μM) on the viability of PC-9 cells for 48 h. Results are represented as means ± standard deviations (n = 5). Statistical significance is indicated as follows: ^*^ *p* < 0.05, ^***^ *p* < 0.001 compared with NC group (Rapa or/and Osi = 0 μM); ^##^ *p* < 0.01, ^###^ *p* < 0.001 compared with Rapa + Osi group. NC, normal control.

As shown in Figures 1A,B, the cell viability of A549 and PC-9 cells gradually decreased with the increase of the concentrations of Rapa and Osi at 24 h and 48 h. The cell viability of A549 and PC-9 cells both were between 50% and 60% when being treated with Rapa (0.5 μM) and Osi (1 μM). Therefore, the Rapa at a concentration of 0.5 μM and the Osi at a concentration of 1 μM were used for subsequent studies. Meanwhile, as illustrated in Figures 1C,D, the cell viability of A549 and PC-9 cells was significantly downregulated in the Rapa and Osi groups compared with the control group. In addition, the cell viability of A549 and PC-9 cells in the Rapa + Osi group was dramatically lower than the Rapa and Osi groups. The CI value calculated by the Chou-Talalay method (CI = 0.06 < 1), indicating that rapamycin combined with osimertinib has a synergistic effect. The above results indicated that Rapa combined with Osi reduced the cell viability of A549 and PC-9 cells.

### 3.2 Rapamycin combined with osimertinib suppressed the proliferation of A549 and PC-9 cells

**FIGURE 2:**
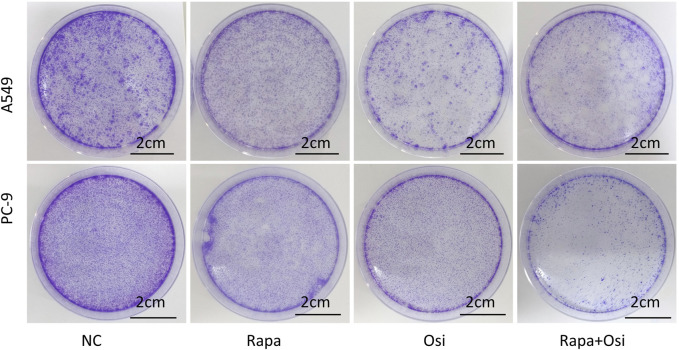
Colony formation assay showing the inhibitory effects of Rapa or/and Osi on the proliferation of A549 and PC-9 cells. The A549 and PC-9 cells were seeded in round cell culture plates at a density of 800 cells plates and treated with Rapa (0.5 μM) or/and Osi (1 μM), incubated for 14 days to allow colony formation. The images of cell colonies formed by A549 and PC-9 cells after treatment with Rapa, Osi, or Rapa + Osi. NC, normal control. Scale bar = 2 cm.

To further affirm their effects on the cell viability of A549 and PC-9 cells, we investigated the effects of Rapa combined with Osi on the proliferation of A549 and PC-9 cells. As illustrated in Figure 2, the number of cell clones of A549 and PC-9 cells was prominently downregulated in the Rapa and Osi groups, compared with the control group. Meanwhile, the number of cell clones of A549 and PC-9 cells was further reduced when treated with the combination of Rapa and Osi, compared with the Rapa or Osi group. The above results demonstrated that Rapa combined with Osi suppressed the proliferation of A549 and PC-9 cells.

### 3.3 Rapamycin combined with osimertinib inhibited the migration of A549 and PC-9 cells

**FIGURE 3:**
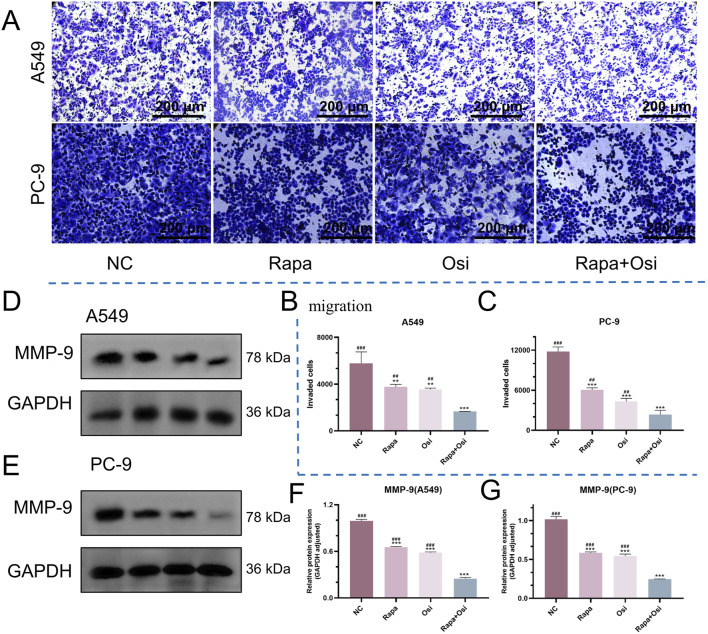
Inhibitory effects of Rapa and Osi on the migration and invasion of A549 and PC-9 tumor cells. The A549 and PC-9 cells were treated with Rapa (0.5 μM) or/and Osi (1 μM) for 48 h, and the cell protein were extracted for western blot experiments. Cells are treated with the same concentration of drug and cultured for 24 h for transwell migration assays. **(A)** Representative images from transwell migration assays showing the number of migrated cells in different treatment groups: Rapa, Osi, and Rapa + Osi. **(B, C)** Quantitative analysis of migrated cells in A549 and PC-9 cell lines, respectively. **(D, E)** Western blot analysis of MMP-9 protein expression in A549 and PC-9 cells under different treatments. GAPDH was used as the loading control. **(F, G)** Quantitative analysis of MMP-9 protein expression normalized to GAPDH in A549 and PC-9 cells, respectively. Results are represented as means ± standard deviations for at least three independent experiments. Statistical significance is indicated as follows: ^**^ *p* < 0.01, ^***^ *p* < 0.001 compared with NC group; ^##^ *p* < 0.01, ^###^ *p* < 0.001 compared with Rapa + Osi group. NC, normal control. Scale bar = 200 μm.

After the effects of Rapa combined with Osi on the cell proliferation of A549 and PC-9 cells have been confirmed, we next investigated the effects of Rapa combined with Osi on the migration of A549 and PC-9 cells using the Transwell, qRT-PCR, and Western blot approaches. As presented in Figures 3A–G, the number of migrated A549 and PC-9 cells and the MMP-9 mRNA and protein expression levels of A549 and PC-9 cells in the Rapa or Osi group were substantially lower than those in the control group. Meanwhile, the number of migrated A549 and PC-9 cells and the MMP-9 mRNA and protein expression levels of A549 and PC-9 cells were further reduced when being treated with the combination of Rapa and Osi. The above results suggested that Rapa combined with Osi inhibited the migration of A549 and PC-9 cells.

### 3.4 Rapamycin combined with osimertinib regulated the cell cycle of A549 and PC-9 cells

**FIGURE 4:**
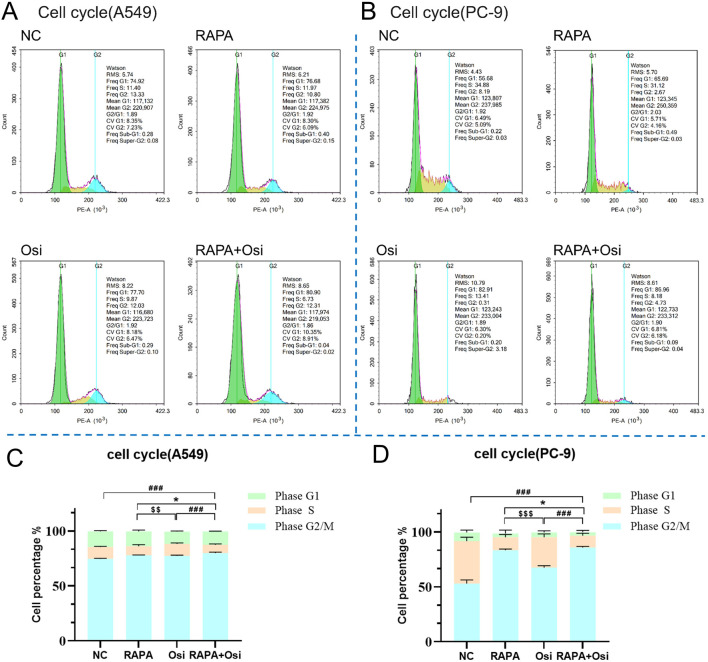
Effects of Rapa or/and Osi on the cell cycle distribution of A549 and PC-9 tumor cells. A549 and PC-9 cells were treated with the Rapa (0.5 μM) or/and Osi (1 μM) for 48 h, then fixed in 70% ethanol and stained with propidium iodide (PI). **(A, B)** Representative flow cytometry histograms showing cell cycle distribution in A549 and PC-9 cells treated Rapa, Osi, or Rapa + Osi. **(C, D)** Quantitative analysis of cell cycle distribution (G1, S, and G2/M phases) in A549 and PC-9 cells with different treatments. Results are represented as means ± standard deviations (n = 3). Statistical significance for S phase is indicated as follows: ^*^ *p* < 0.05 compared RAPA with RAPA + Osi group; ^$$^ *p* < 0.01, ^$$$^ *p* < 0.001 compared RAPAwith Osi group; ^###^ *p* < 0.001 compared with Rapa + Osi group. NC, normal control.

As the effects of Rapa combined with Osi on the progression of A549 and PC-9 cells have been affirmed in the above, we subsequently investigated the effects of Rapa combined with Osi on the cell cycle of A549 and PC-9 cells. As illustrated in Figures 4A–D, Rapa group significantly decreased and the percentage of the S phase of A549 and PC-9 cells, and Osi increased the effect of Rapa. In addition, the percentage of the S phase of A549 and PC-9 cells in the Rapa group was prominently lower than the Osi group, indicating that Rapa plays a major role in blocking the cell cycle. The above results affirmed that Rapa combined with Osi blocks A549 and PC-9 cells in the G1 phase, and fewer cells enter the S phase.

### 3.5 Rapamycin combined with osimertinib promoted the apoptosis of A549 and PC-9 cells

**FIGURE 5:**
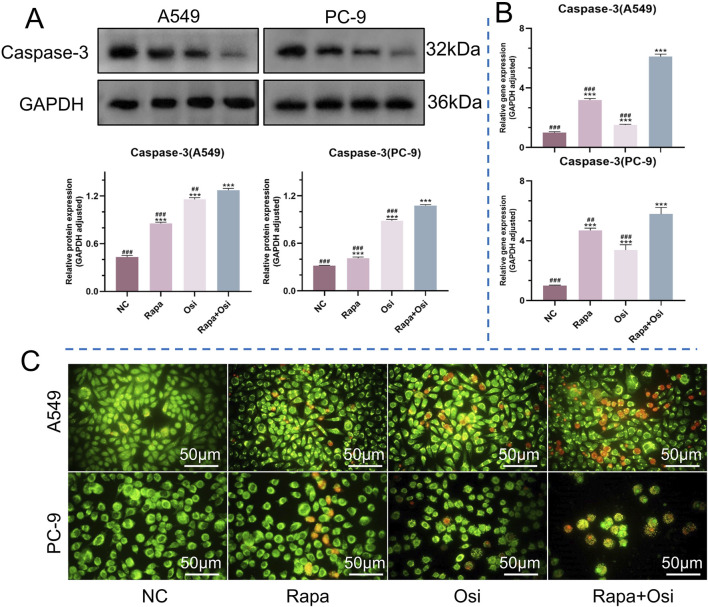
Effects of Rapa or/and Osi on apoptosis in A549 and PC-9 tumor cells. The A549 and PC-9 cells were treated with Rapa (0.5 μM) or/and Osi (1 μM) for 48 h, and the cell protein were extracted for western blot experiments. Cells were incubated with Rapa (0.5 μM) or/and Osi (1 μM) for 48 h to observe cell the apoptosis of A549 and PC-9 cells. **(A)** Western blot analysis of caspase-3 protein expression in A549 and PC-9 cells treated with NC, Rapa, Osi, or Rapa + Osi. GAPDH was used as the loading control. Quantitative analysis of caspase-3 expression normalized to GAPDH is also shown. **(B)** Relative mRNA expression levels of caspase-3 in A549 and PC-9 cells determined by qPCR. **(C)** AO/EB (Acridine Orange/Ethidium Bromide) staining showing morphological changes in A549 and PC-9 cells indicative of apoptosis under different treatments. Green fluorescence indicates live or early apoptotic cells, while red fluorescence represents late apoptotic or necrotic cells. Results are represented as means ± standard deviations (n = 3). Statistical significance is indicated as follows: ^***^ *p* < 0.001 compared with NC group; ^##^ *p* < 0.01, ^###^ *p* < 0.001 compared with Rapa + Osi group. NC, normal control. Scale bar = 50 μm.

**FIGURE 6:**
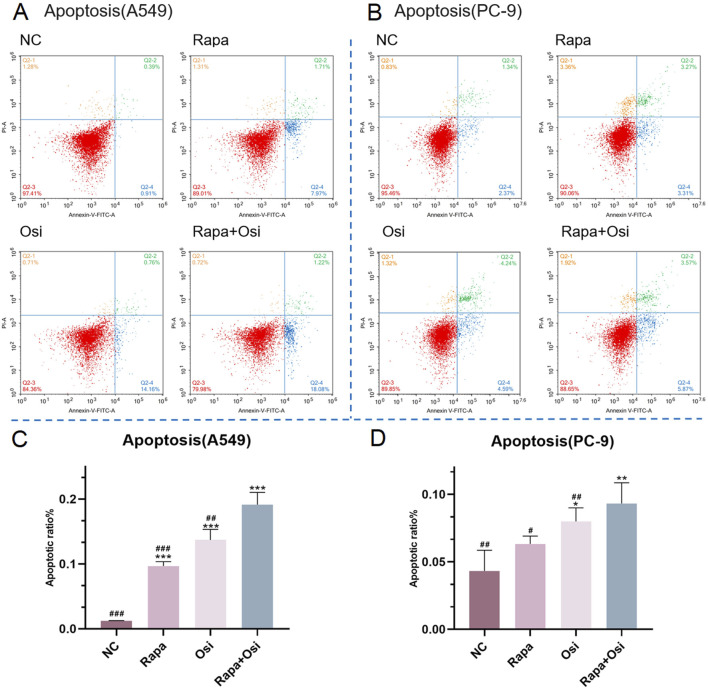
Effects of Rapa and Osi on apoptosis in A549 and PC-9 tumor cells. A549 and PC-9 cells were treated with Rapa (0.5 μM) or/and Osi (1 μM) for 48 h, then incubated with Annexin V-FITC and PI. **(A, B)** Representative flow cytometry dot plots using Annexin V/PI staining to assess apoptotic cell distribution in A549 and PC-9 cells treated with NC, Rapa, Osi, or Rapa + Osi. The quadrants indicate Q2-1: necrotic cells (Annexin V-/PI+), Q2-2: late apoptotic cells (Annexin V+/PI+), Q2-3: live cells (Annexin V-/PI-), and Q2-4: early apoptotic cells (Annexin V+/PI-). **(C)** Quantitative analysis of the percentage of apoptotic cells (early apoptosis) in A549 cells. **(D)** Quantitative analysis of the percentage of apoptotic cells (early apoptosis) in PC-9. Results are represented as means ± standard deviations (n = 3). Statistical significance is indicated as follows: ^*^ *p* < 0.05, ^**^ *p* < 0.01, ^***^ *p* < 0.001 compared with NC group; ^#^ *p* < 0.05, ^##^ *p* < 0.01, ^###^ *p* < 0.001 compared with Rapa + Osi group. NC, normal control.

The effects of Rapa combined with Osi on the apoptosis of A549 and PC-9 cells were further investigated using flow cytometry, qRT-PCR, and Western blot approaches. As shown in Figures 5, 6, compared with the control group, the apoptosis rate, the mRNA expression and protein expression level of Caspase-3 of A549 and PC-9 cells in the Rapa or Osi group were significantly upregulated. In addition, the apoptosis rate, the mRNA and protein expression level of Caspase-3 of A549 and PC-9 cells in the Rapa + Osi group were dramatically higher than those in the Rapa or Osi group. The above results suggested that Rapa combined with Osi promoted the apoptosis of A549 and PC-9 cells.

### 3.6 Rapamycin combined with osimertinib increased the ROS level of A549 and PC-9 cells

**FIGURE 7:**
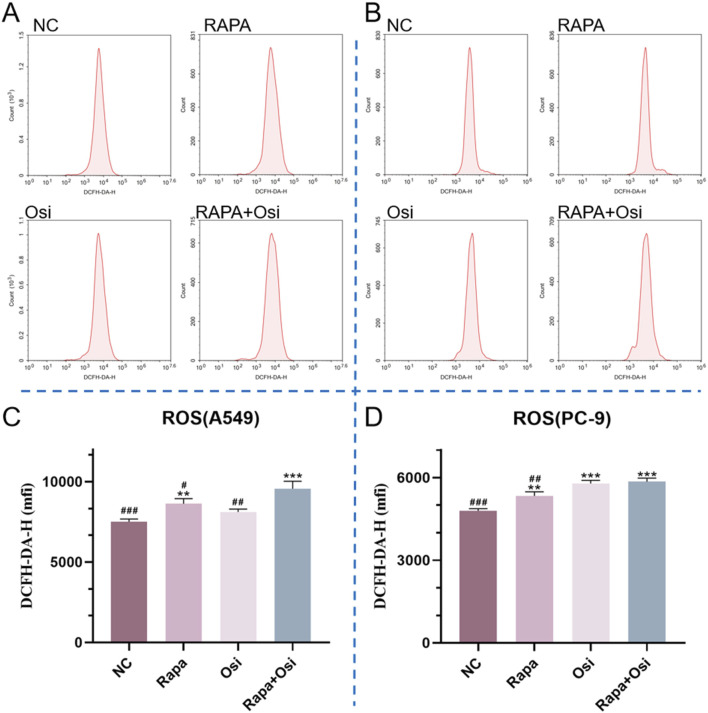
Effects of Rapa and Osi on ROS levels in A549 and PC-9 cells. The cells were treated with the Rapa (0.5 μM) or/and Osi (1 μM) for 48 h, and then diluted DCFH-DA was added to the cells, which were then incubated in a 37°C incubator for 20 min **(A, B)** The flow cytometry plots showing ROS levels in A549 and PC-9 cells after treatment with Rapa, Osi, or Rapa + Osi, using the ROS Fluorometric Assay Kit (Red). The x-axis represents cell count, and the y-axis shows DCFH-DA-H fluorescence intensity, which correlates with ROS levels in the cells. **(C)** Quantitative analysis of ROS levels in A549 cells. **(D)** Quantitative analysis of ROS levels in PC-9 cells. Results are represented as means ± standard deviations (n = 3). Statistical significance is indicated as follows: ^**^ *p* < 0.01, ^***^ *p* < 0.001 compared with NC group; ^#^ *p* < 0.05, ^##^ *p* < 0.01, ^###^ *p* < 0.001 compared with Rapa + Osi group. NC, normal control.

As the activation of oxidative stress is one of the significant therapeutic mechanisms for NSCLC, we next investigated the effects of Rapa combined with Osi on the ROS level of A549 and PC-9 cells (Rosell et al., 2023). As illustrated in Figures 7A–D, compared with the control group, the ROS level of A549 and PC-9 cells in the Rapa or Osi group were prominently upregulated. In addition, Rapa combined with Osi further upregulated the ROS level of A549 and PC-9 cells. The above results suggested that Rapa combined with Osi increased the ROS level of A549 and PC-9 cells.

### 3.7 Rapamycin combined with osimertinib promoted autophagy of A549 and PC-9 cells

**FIGURE 8:**
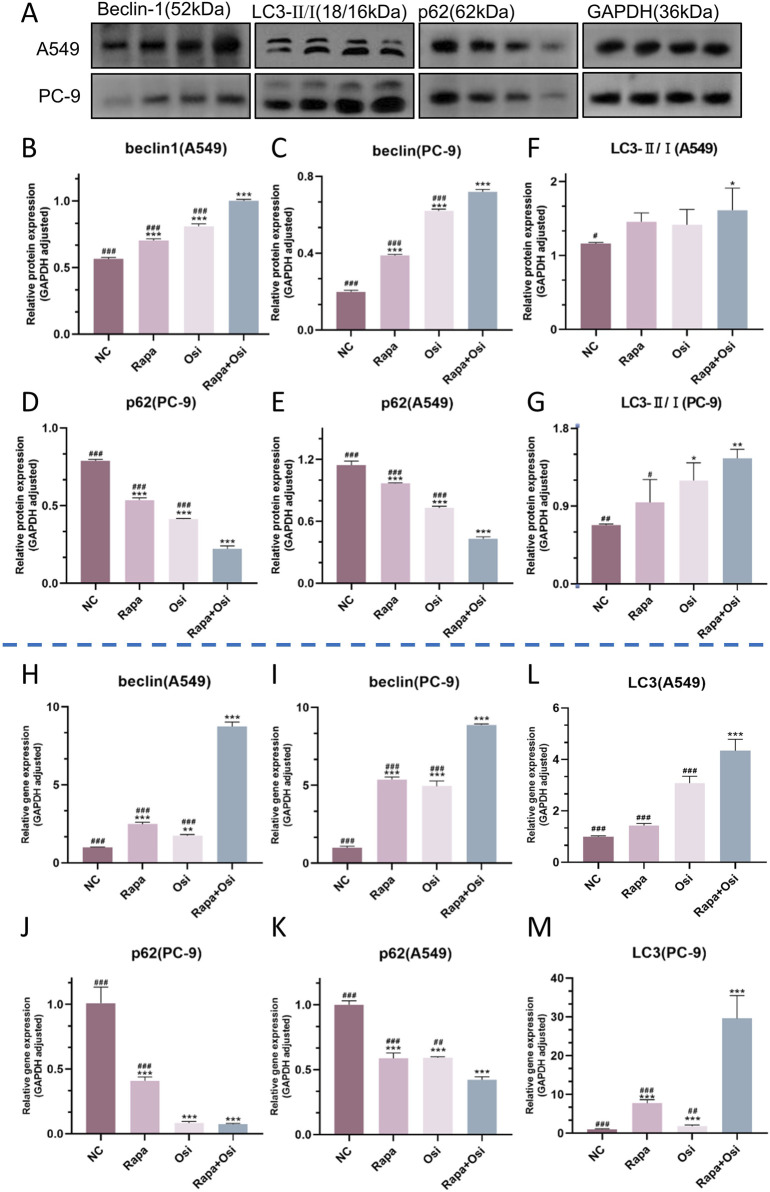
Effects of Rapa and Osi on autophagy in A549 and PC-9 cells. The A549 and PC-9 cells were treated with Rapa (0.5 μM) or/and Osi (1 μM) for 48 h, and the cell protein were extracted for western blot experiments. **(A)** The western blot images showing the expression of beclin1, p62 and LC3 II/I in A549 and PC-9 cells, with GAPDH as a loading control. **(B–G)** Quantitative analysis of the relative expression levels of beclin1, p62 and LC3 II/I in A549 and PC-9 cells after treatment with Rapa, Osi, or Rapa + Osi. **(H**–**M)** Gene expression analysis of beclin1, p62 and LC3 II in A549 or PC-9 cells determined by qPCR. Results are represented as means ± standard deviations (n = 3). Statistical significance is indicated as follows: ^*^ *p* < 0.05, ^**^ *p* < 0.01, ^***^ *p* < 0.001 compared with NC group; ^#^ *p* < 0.05, ^##^ *p* < 0.01, ^###^ *p* < 0.001 compared with Rapa + Osi group. NC, normal control.

As autophagy plays an essential role in the development of NSCLC, we further investigated the effects of Rapa and Osi on the autophagy of A549 and PC-9 cells (Liao et al., 2023). As presented in Figures 8A–M, the tendency of the mRNA expression level of LC3, the protein expression level of LC3 II/I, and the mRNA and protein expression levels of beclin1 were opposite to the mRNA and protein expression levels of p62. Compared with the control group, the mRNA expression level of LC3, the protein expression level of LC3 II/I, and the mRNA and protein expression levels of beclin1 in A549 and PC-9 cells of the Rapa or Osi group were significantly upregulated, and the mRNA and protein expression levels of p62 in A549 and PC-9 cells of the Rapa or Osi group were dramatically downregulated. In addition, the combined administration of Rapa and Osi further reduced the mRNA and protein expression levels of p62 and elevated the mRNA expression level of LC3, the protein expression level of LC3 II/I, and the mRNA and protein expression levels of beclin1 in A549 and PC-9 cells. The above results declared that Rapa combined with Osi promoted the autophagy of A549 and PC-9 cells.

### 3.8 Rapamycin combined with osimertinib regulated the PARP, Akt/mTOR, and MAPK/ERK signaling of A549 and PC-9 cells

**FIGURE 9:**
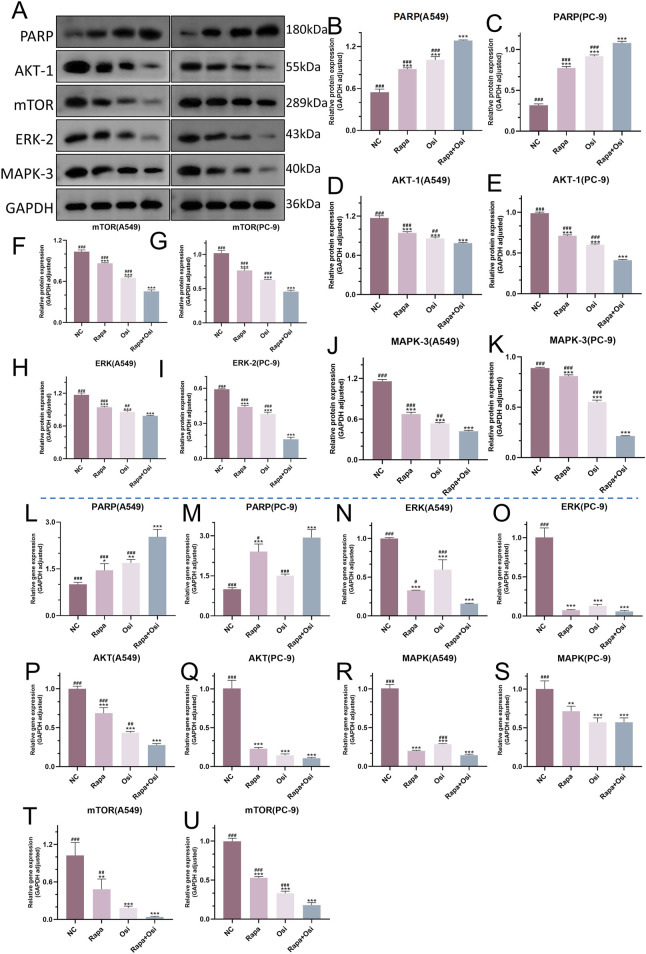
Effects of Rapa and Osi on the regulation of PARP, Akt/mTOR, and MAPK/ERK signaling pathways in A549 and PC-9 cells. A549 and PC-9 cells was treated with Rapa, Osi, or Rapa + Osi. The A549 and PC-9 cells were treated with Rapa (0.5 μM) or/and Osi (1 μM) for 48 h, and the cell protein and mRNA were extracted for western blot and qRT-PCR experiments. **(A)** The western blot images showing the protein expression levels of PARP, AKT-1, mTOR, ERK-2, and MAPK-3 in A549 and PC-9 cells, with GAPDH used as a loading control. **(B–K)** Quantitative analysis of PARP, AKT-1, mTOR, ERK-2, and MAPK-3 protein levels in A549 or PC-9 cells normalized to GAPDH. **(L-U)** Gene expression analysis of PARP, AKT, mTOR, ERK, and MAPK in A549 or PC-9 cells measured by qRT-PCR. Results are represented as means ± standard deviations (n = 3). Statistical significance is indicated as follows: ^*^ *p* < 0.05, ^**^ *p* < 0.01, ^***^ *p* < 0.001 compared with NC group; ^#^ *p* < 0.05, ^##^ *p* < 0.01, ^###^ *p* < 0.001 compared with Rapa + Osi group. NC, normal control.

As the effects of Rapa combined with Osi on the progression, ROS level, and autophagy of A549 and PC-9 cells have been confirmed in the above, we subsequently investigated the related mechanisms of Rapa combined with Osi on A549 and PC-9 cells. As shown in Figures 9A–U, the tendency of the mRNA and protein expression level of Akt, mTOR, MAPK, and ERK was contrary to the mRNA and protein expression level of PARP. Compared with the control group, the mRNA and protein expression level of Akt, mTOR, MAPK, and ERK in A549 and PC-9 cells of the Rapa or Osi group were prominently downregulated, and the mRNA expression level of PARP and the protein expression level of PARP in A549 and PC-9 cells of the Rapa or Osi group were memorably upregulated. In addition, the combined administration of Rapa and Osi further downregulated the mRNA and protein expression level of Akt, mTOR, MAPK, and ERK and upregulated the mRNA expression level of PARP and the protein expression level of PARP in A549 and PC-9 cells. The above results demonstrated that Rapa combined with Osi activated the PARP signaling pathway and inhibited the Akt/mTOR and MAPK/ERK signaling pathways.

## 4 Discussion

Targeted therapy is routinely recommended for treating NSCLC patients carrying ECRF mutations. Although three generations of EGFR-TKI have been approved for treating NSCLC patients carrying ECFR mutations in clinical practice, acquired drug resistance inevitably occurs in patients receiving EGFR-TKI, such as Osi (Wu and Shih, 2018). In recent years, studies have found that the mTOR signaling pathway is closely related to the formation of drug resistance for NSCLC (Gargalionis et al., 2023). However, the method of Rapa to overcome the Osi resistance in NSCLC patients is unknown. Therefore, this study investigated whether Rapa combined with Osi to exert synergistic effects in treating A549 and PC-9 cells and judged the potential role of Rapa overcomes Osi resistance in NSCLC patients.

In the present study, we first the optimal administrated concentrations of Rapa and Osi on A549 and PC-9 cells by CCK-8. As the cell viability of A549 and Osi both were between 50% and 60%, Rapa (0.5 μM) and Osi (1 μM) were used for subsequent studies. Meanwhile, our results also demonstrated that Rapa combined with Osi further downregulated the cell viability of A549 and PC-9 cells compared with the single administration of Rapa or Osi. In recent years, previous studies have affirmed that Rapa reversed cisplatin resistance in lung cancer to affect its progression, which is associated with the mTOR-mediated signaling pathway (Wangpaichitr et al., 2008; Wu et al., 2005). Rapa also induce apoptosis by modulating Bcl-2, P53 and Bax gene expression, and increasing intracellular Ca^2+^ concentration, and the apoptosis induction effect have timeliness and dose-effect (Cao and Ma, 2020). Meanwhile, Rapa prevented cell migration by inhibiting the expression of MMP9 (Gao et al., 2011). In addition, it was demonstrated Rapa inhibited the expression of cell cycle proteins and the activity of cell cycle-dependent kinases to block the G1 to S phase of the cell cycle via specifically binding to FKBP to prevent downstream phosphorylation of S6K1 and 4E-BP1 (Chen et al., 2015). Therefore, we subsequently investigated the effects of Rapa combined with Osi on the progression of A549 and PC-9 cells including proliferation, migration, apoptosis, and cell cycle. In this study, we demonstrated that the proliferation and migration of cells as well as the mRNA and protein expression levels of MMP9 were significantly decreased in the Rapa or Osi group, and the above indicators further reduced when treated with the combination of Rapa and Osi. Meanwhile, the apoptosis rate, the mRNA expression level of Caspase-3, and the protein expression level of cleaved Caspase-3/Caspase-3 were all dramatically upregulated in the Rapa or Osi group, and Rapa combined with Osi further increased the above indicators. In addition, the results showed that Rapa or Osi downregulated the percentage of the S phase, and the alterations was promoted by the treatment of Rapa combined with Osi and indicated the growth cycle of the 549 and PC-9 cells was blocked in the G1 phase. The effects of Rapa combined with Osi reduced cell viability, proliferation, and migration, promoted apoptosis, and regulated the cell cycle of A549 and PC-9 cells were demonstrated, which were consistent with the previous study mentioned above. ROS is an essential part of the signal transduction mechanism in cancer cells, whose increase induces autophagy (Wang et al., 2023). ROS further inhibited the Akt/mTOR and MAPK/ERK signaling pathways in cancer cells (Fang et al., 2021; Jia et al., 2021). Resveratrol, as an activator of SIRT1, increased the expression of LC3 II/I and beclin1, decreased p62 expression, and induced the autophagy of NSCLC cells by suppressing the Akt/mTOR signaling pathway (Wang et al., 2018). Meanwhile, another study demonstrated that chrysoeriol upregulated the expression of LC3 II and beclin1 and induced autophagy of A549 cells by inhibiting the MAPK/ERK signaling pathway (Wei et al., 2019). These pathways are potent directions for the study of NSCLC. In addition, the previous studies also affirmed that Rapa and Osi alleviated lung cancer by activating autophagy (Bi et al., 2021; Wang et al., 2024). Therefore, we next investigated whether Rapa combined Osi mitigated NSCLC increased the ROS level to regulate the Akt/mTOR and MAPK/ERK signaling pathways, thereby inducing autophagy. Based on the above studies, we design experiments and affirmed that the single administration of Rapa or Osi prominently upregulated the ROS level, the protein and mRNA expression level of LC3 II/I and beclin1, and downregulated the protein and mRNA expression in pathway of MAPK, ERK, Akt, mTOR, and p62. Meanwhile, Rapa combined with Osi can further increase the ROS level, inhibited the MAPK/ERK and Akt/mTOR signaling pathways, and activated autophagy.

In addition, PARP exerts its cytotoxicity in the clinical treatment of cancer by inhibiting PARP polymerase and capturing PARP-DNA (Chan et al., 2021). Toxin microcystin from Cyanobacteria upregulated the PARP pathway to alleviate NSCLC, and anti-tumor xanthones from Garcinia nujiangensis inhibited the MAPK/ERK and Akt/mTOR signaling pathways and activated the PARP pathway to suppress proliferation and induce apoptosis of ovarian cancers cells (Apopa et al., 2018; Tang et al., 2020). Therefore, we also investigated whether Rapa combined with Osi regulated the PARP pathway in A549 and PC-9 cells. Our results presented that Rapa combined with Osi substantially increased the protein and mRNA expression in PARP pathway, with better performance compared to Rapa or Osi alone. All in all, the regulation of the PARP, MAPK/ERK, and Akt/mTOR signaling pathways were the key factors of Rapa combined with Osi to alleviate NSCLC.

The results provided evidence of cell level of the combination of Rapa and Osi in the treatment of NSCLC and an important theoretical basis for clinical practice, but the clinical application needs to further consider dose optimization, patient-specific factors, and combination therapy strategies. Based on experimental results existing clinical data, the rational initial regimen of Osi (80 mg/day) with Rapa (2.5–5 mg/day) is recommended with adjustments by patient-specific factors such as age, renal function, and metabolic status (Corbacioglu et al., 2024; Cheng et al., 2021). At the same time, based on the principle of the combination of Rapa and Osi, similar drugs can be selected for clinical trials to obtain better drug combinations. The experimental results showed that the combination of Rapa and Osi significantly inhibited the Akt/mTOR and MAPK/ERK signaling pathways. Clinically, treatment response and resistance can be assessed by measuring the activity of these pathways, such as phosphorylation levels.

However, there are still some shortcomings in the present study. First of all, although the synergistic effects of Rapa and Osi in treating A549 and PC-9 cells have been affirmed, it is still uncertain whether Rapa overcomes the Osi resistance in NSCLC. Therefore, we consider it urgent to conduct more experiments to investigate the effects and related mechanisms of Rapa in overcoming Osi resistance. Moreover, our study lacks animal experiments to demonstrate the therapeutic effect of Rapa combined with Osi in the body, and the next phase of our work is complemente the animal experiments to investigate the effects and related mechanisms of Rapa combined with Osi *in vivo*. In addition, ferroptosis, mitophagy, and Nrf2 transcriptional factor is related to Rapa and Osi can be conducted in subsequent studies.

In summary, the study has established the efficacy and related mechanisms of the combination of Rapa and Osi in alleviating NSCLC and laied the groundwork for future research on overcoming Osi-resistant NSCLC patients with Rapa, but additional studies should be warranted to further elucidate the therapeutic mechanisms of the Rapa-Osi combination in treating NSCLC. This would offer enhanced theoretical support for the treatment of NSCLC through the use of Rapa combined with Osi.

## 5 Conclusion

Taken together, this study demonstrated that Rapa combined with Osi reduced cell viability, proliferation, and migration, regulated the cell cycle, and increased the ROS level, apoptosis, and autophagy of A549 and PC-9 cells by activating the PARP pathway and inhibiting the MAPK/EKR and Akt/mTOR pathways, providing a novel theoretical basis for their clinical treatment of NSCLC.

## Data Availability

The raw data supporting the conclusions of this article will be made available by the authors, without undue reservation.
